# Modulating the Fatty Acid Profiles of *Hermetia illucens* Larvae Fats by Dietary Enrichment with Different Oilseeds: A Sustainable Way for Future Use in Feed and Food

**DOI:** 10.3390/insects13090801

**Published:** 2022-09-01

**Authors:** Bogdan Georgescu, Anca Mihaela Boaru, Leon Muntean, Nicușor Sima, Dănuț Ioan Struți, Tudor Andrei Păpuc, Carmen Georgescu

**Affiliations:** 1 Department of Zoology and Ecology, Faculty of Animal Science and Biotechnologies, University of Agricultural Sciences and Veterinary Medicine of Cluj-Napoca, 400372 Cluj-Napoca, Romania; 2 Department of Plant Culture, Faculty of Agriculture, University of Agricultural Sciences and Veterinary Medicine of Cluj-Napoca, Calea Mănăştur 3-5, 400372 Cluj-Napoca, Romania; 3 Department of Technological Science, Faculty of Animal Science and Biotechnologies, University of Agricultural Sciences and Veterinary Medicine of Cluj-Napoca, 400372 Cluj-Napoca, Romania; 4 Department of Endocrinology, “Iuliu Hatieganu” University of Medicine and Pharmacy, 400349 Cluj-Napoca, Romania

**Keywords:** oil seeds, fat quality, omega–3, dietary modulation, rearing substrate

## Abstract

**Simple Summary:**

Most of the vegetable oils represent a suitable source of unsaturated fatty acids and bioactive compounds, which can be the key to solving different nutritional limitations. The fatty acid profile of *Hermetia illucens* larvae fats can be modulated through dietary enrichment as a result of adding vegetable oils to the rearing substrate. Therefore, the present research analyzes the effects of a 10% addition of vegetable oils from five dietary fat sources in larvae diets on the productive performances and fatty acid profiles of *H. illucens*. Oil inclusion in the larval diet improved (*p* < 0.05) the weight of larvae, prepupae, pupae, and imago without influencing (*p* > 0.05) the egg clutch weight and the number of eggs in the clutch. In addition, the larvae FA profile was enhanced in unsaturated fatty acids (*p* < 0.001), especially in long-chain polyunsaturated fatty acids from the omega-3 series, when the linseed oil, hempseed oil, and rapeseed oil were used in larvae diets.

**Abstract:**

Edible insects such as the black soldier fly *Hermetia illucens* L. represent a potential and sustainable source of nutrients for food and feed due to their valuable nutritional composition, which can be modulated through dietary enrichment. The high content of saturated fatty acid (FA) of *Hermetia illucens* larvae fats can be modulated through dietary enrichment as a result of adding vegetable oils in the rearing substrate. Therefore, the present research aims to highlight the effects of a 10% addition of vegetable oils from five dietary fat sources (linseed oil, soybean oil, sunflower oil, rapeseed oil, and hempseed oil) on the growth, development, reproductive performance, and the fat and fatty acids profile of *H. illucens*. Oil inclusion in the larval diet improved (*p* < 0.05) the weight of larvae, prepupae, pupae, and imago without influencing (*p* > 0.05) the egg clutch weight and the number of eggs in the clutch. In addition, the larvae fatty acid profile was different (*p* < 0.001) according to the oil type, because the unsaturated FAs (UFA) increased from 11.23 to 48.74% of FAME, as well as according to the larvae age, because the saturated FAs decreased from 85.86 to 49.56% of FAME. Linseed oil inclusion led to the improvement of the FA profile at 10 days age of larvae, followed by hempseed and rapeseed oil. These three dietary treatments recorded the highest concentrations in UFA (29.94–48.74% of FAME), especially in polyunsaturated FA (18.91–37.22% of FAME) from the omega-3 series (3.19–15.55% of FAME) and the appropriate n–6/n–3 ratio. As a result, the degree of the lipid polyunsaturation index increased (17.76–41.44) and the value of the atherogenic (3.22–1.22) and thrombogenic (1.43–0.48) indices decreased. Based on the obtained results, it can be concluded that enriching the larval diet with these oils rich in UFA can modulate the larvae FA profile, making them suitable sources of quality fats for feed and indirectly for food.

## 1. Introduction

Vegetable oils from the seeds of various plants such as flax, soybean, hemp, and rape represent a common and proper source of unsaturated fatty acids [1,2,3,4]. The beneficial properties for human health and animal health of long-chain polyunsaturated fatty acids available in plants are also known [5,6,7,8]. Insects are increasingly used as a source of valuable nutrients for animal feed, but also for various medical issues or for biodiesel [9,10]. In the context of sustainable development, current technological possibilities could allow the eco-friendly rearing of insects, thus becoming a sustainable solution to provide future high-quality food and feed [11,12,13,14,15]. Among the insect species, attention is frequently focused on the potential of *Hermetia illucens* L. (Diptera: Stratiomyidae, 1758), commonly known as black soldier fly (BSF) [16].

In order to meet the increasing demand of fish (rich in omega-3 fatty acids) for human food, global aquaculture has experienced great development, requiring large quantities of fishmeal and fish oil [17]. *Hermetia illucens* larvae have gained the attention of numerous research studies as alternative sources of nutrients for aquafeeds and are already regulated by the EU (2017/893/EC) for use in aquaculture feeds if they are obtained from vegetable substrates [18]. For this reason, the rearing substrate has proven to play a crucial role, together with the exclusive feeding in the larval stage [16]. The high fat content of larvae and prepupae (20–40%), as well as the high content of saturated fatty acids (SFA—over 60% of FAME), are some of the factors limiting their use in animal feed [19,20,21]. In this regard, two alternatives are now available: meal defatting, or adjustment of the larvae fatty acid profile by dietary enrichment [21,22,23,24]. Defatting is known and involves a technological process with additional costs [25,26]. Previous studies have demonstrated the major influence of the rearing substrate on the nutrient content of larvae, thus providing a background for the useful modulation of the larvae nutrient profile [27,28,29]. Usually, the omega-3 (n–3) FA concentration in the rearing diets of *H. illucens* larvae is low; therefore, the larval profile is poor in n–3 FA [30,31,32]. Fish by-products have been used for the enrichment of larval rearing substrate [21,33], and only few studies are focused on the utilization of vegetable origin sources rich in polyunsaturated fatty acids (PUFAs) to improve the substrate [24,34,35]. In this regard, Oonincx et al. [36] report an improvement in the fatty acid profile of the *Hermetia illucens* larvae fats as a result of a 1–4% addition of flaxseed oil (rich in n-3 fatty acids) in the larvae-rearing diets. Recently, the progressive enrichment (10–100%) of the larvae diets (based on chicken feed) with flax and rape cakes has led to a progressive increase of unsaturated fatty acids (UFA), together with the decrease of SFA in prepupae fats [34].

Based on this background, we hypothesized that if different types of vegetable oils rich in UFA are incorporated in larvae diets, the fatty acid profile of larvae fats could be enriched during larval development. Thus, the aim this research was to demonstrate the effects of adding vegetable oils to larvae diets on the fatty acid profile of larval fats, growth, and reproduction performances of *Hermetia illucens*.

## 2. Materials and Methods

### 2.1. Study Site and Biological Material Origin

The study was carried out at the Faculty of Animal Science and Biotechnologies, University of Agricultural Sciences and Veterinary Medicine Cluj-Napoca, Romania, in 2019. The biological material came from a colony of *Hermetia illucens* maintained year-round in an indoor laboratory for 6 years (with a mean number of 5.5 generations per year). The initial population of *Hermetia illucens* was purchased from a Greek breeding farm. Optimal medial parameters were ensured in the colony (27 ± 0.3 °C; 65 ± 10 RH; 16 h photoperiod natural light supplemented with LED light—6000 lux) [37,38]. Six egg clutches with similar weights (23.27–25.02 mg) were collected from eggs laid within 24 h, each being introduced in an experimental diet.

### 2.2. Evaluated Parameters

The study recorded the effects of adding vegetable oils as a source of unsaturated fatty acids in the larvae-rearing diets on the: (1) larvae crude fat content and evolution of the fatty acid profile of larval fats at different ages; (2) evolution of the body weight of larvae, prepupae, pupae, and adults; and (3) reproductive parameters of flies and egg clutch quality.

### 2.3. Experimental Design

The experimental diets use for larvae rearing were formulated as follows: the Gainesville diet (≈13.5% crude protein and 3.4% crude fat) [39] without oil addition was considered to be the control diet (control group), and for the next five experimental diets (groups), linseed, soybean, sunflower, rapeseed, and hempseed oils were added in an amount of 10% of the dry mass. The Gainesville diet was created for houseflies and consists of 20% corn, 30% alfalfa meal, and 50% wheat bran [39].

The rearing substrate for each experimental diet was properly prepared before egg addition: 70 ± 5% humidity and 26 °C ± 0.4 [16]. Larvae rearing was carried out in dark boxes measuring 33 cm × 23 cm × 13 m (L × L × h). The larvae hatched 50–72 h after egg oviposition. BSF larvae were fed progressively as they consumed the feed. Tert-butylhydroquinone (200 μg/g oil) was added to prevent oil oxidation [36,40]. At the age of 20–21 days, the emergence process associated with the metamorphosis to the prepupal stage occurred from the substrate. During the prepupal and pupal stages, the BSF individuals were maintained on a dry leaf substrate, each group being placed separately in an opaque plastic box measuring 15 cm × 10 cm × 5 cm and with vents, being maintained in the laboratory at a temperature of 27 °C ± 0.5 and a humidity of 65% ± 4 RH [38,41]. BSF flies from each group were kept in the rearing cages measuring 50 cm × 35 cm × 45 cm, ensuring optimal parameters of 27 °C ± 0.1 and 65% ± 2 RH [38,41]. The fly cages were equipped with a plastic container (dimensions 20 cm × 15 cm × 10 cm) with an attractive substrate for oviposition, consisting of 50% wheat bran, 30% alfalfa meal, and 20% corn meal [39]. The box with the attractive substrate was covered with a mosquito net, over which the oviposition support was placed, consisting of 10 overlapping wooden boards with a distance of 3 mm between the boards [42]. The wooden support for egg oviposition was replaced daily with a new sterilized support. The lighting period for flies was 16 h of light (16 h photoperiod natural light supplemented with LED and yellow light—6000 lux) [43].

### 2.4. Growing Performances

To record the evolution of the body weight, the larvae from each diet were randomly weighed (with an Explorer-Pro analytical balance, model EP114C, accuracy 0.01 mg) at 10, 15, and 20 days of age, respectively. When the prepupae left the substrate, the weighing was performed. A new weight assessment was performed at the pupal stage. The weight of the adults was assessed at the time of exitus, when the individuals were randomly selected and individually weighed. Each evaluation was performed on a randomly selected number of 50 individuals.

The weight of the egg clutches, the number of eggs in each clutch and the individual weight of an egg were performed according to the group. The eggs in a clutch were counted with an Alpha model binocular magnifier (zoom 7×–45×) by dispersing the eggs in the clump with 70% ethanol, followed by photographic capture and counting with computer software [43]: ClickMaster2000 1.0. (https://www.thregr.org/~wavexx/software/clickmaster2000).

### 2.5. Fat and Fatty Acid Analysis

The larvae crude fat content (n = 5) was determined at 10, 15, and 20 days age, according to the procedures established by AOAC International [44] for the crude fat extraction by the Soxhlet method (no. 920.39).

The fatty acid content was also performed at 10, 15, and 20 days age from the larvae fats (*n* = 5). Before analysis, 10 g larvae/diet were collected and kept for 24 h on a clean substrate for emptying the digestive tract, followed by an impurities wash with ethanol 70% and storage at −80 °C. Extraction and identification of fatty acid methyl esters (FAME) from *Hermetia illucens* larvae fats were performed by gas chromatography with mass spectrometry detection in accordance with AOAC-969.33 [45] and ISO 3657: 2002, ISO 12966-2: 2011, and ISO 12966-2: 2017. The identification and quantification of FAME consisted of fat saponification (with methanolic sodium hydroxide solution 0.5 mol/L on a sand bath at 210 °C and a reflux rate of 1 drop/s up to 30 min after the complete removal of fat traces), followed by esterification under boron trifluoride catalyst 15% vol, and addition of cooling hexane. The equipment used was a Perkin Elmer chromatographic system with a mass spectrometer detector (GC–MS) consisting of a Clarus 680 gas chromatograph and a Clarus SQ8T quadrupole mass spectrometer. The following were used: Elite-Wax chromatographic column with stationary polar phase polyethylene glycol (PEG), length of 30 m, internal diameter of 0.25 mm, and film thickness of 1.0 μm; injection port temperature of 220 °C, injected sample volume of 1.0 μL, helium carrier gas flow rate of 1.5 mL/min, and splitting ratio of 40:1. The operating parameters of the MS were: transfer line temperature of 150 °C, source temperature of 150 °C, multiplier 1500, and solvent delay of 0–1.5 min. The determination of fatty acid concentration was performed by comparing the relative retention time of FAME with that of the certified standard—Mix FAME Supelco 37. The individual fatty acid concentration was expressed in % of the total identified FAME.

Calculating the ratio between the total saturated fatty acids (Σ SFA), total monounsaturated (Σ MUFA), total polyunsaturated (Σ PUFA), and total unsaturated fatty acids (Σ UFA) was performed as follows: Σ PUFA/Σ SFA; Σ MUFA/Σ SFA; Σ UFA/Σ SFA; n-6 (fatty acids from n-6 series); n-3 (fatty acids from n-3 series).

The polyunsaturation index (PI) of fats was calculated according to the equation proposed by Timmons [46]:

PI = C18: 2 n-6 + (C18: 3 n-3 × 2)(1)

The atherogenic index (AI) and thrombogenic index (TI) of lipids were calculated according to the equations proposed by Ulbricht et al. [47]:

AI = (C12:0 + C16:0 + 4 × C14:0) ÷ [ΣMUFA + Σ (n-6) + Σ (n-3)](2)

TI = (C14:0 + C16:0 + C18:0) ÷ [0.5 × ΣMUFA + 0.5 × Σ (n-6) +3 × Σ (n-3) + Σ (n-3) ÷ Σ (n-6)](3)

### 2.6. Statistical Analysis

The differences between the weight of larvae, prepupae, pupae, imago, quality parameters of clutches (total weight, egg number, egg weight), larvae crude fat content, and fatty acid concentrations of fats evaluated between the treatments were subjected to an ANOVA single-way test at a significance level of 5%, followed by the Tukey HSD as a post hoc test (*p* < 0.05). The data were presented as Mean ± SE (standard error).

## 3. Results

### 3.1. Growing Performances

The addition of the vegetable oils in the diets of *Hermetia illucens* larvae led to an increase of the larvae weight compared to the control group (Table 1).

At 10 days of age, a heavier weight of larvae was recorded in the hempseed oil treatment (*p* < 0.05), followed by those in the treatments with sunflower, soybean, and linseed oil (Table 1). In all diets, the larvae weight at 15 days age was higher than the weight recorded at 10 days, and diets with linseed oil provided the heaviest weight of larvae, followed by the diet with soybean oil. At 20 days age of larvae, the body weight decreased compared to the previous assessment only in the diets where vegetable oil was added (Table 1). Insignificant differences in weight compared to the control (*p* > 0.05) were recorded between the larvae of diets with linseed oil and soybean oil. The larval stage was shorter by 3 days for all diets with vegetable oils, which indicated a more intense growing rate and explained the lower weight of the larvae at 20 days age, most of them having been molted and having passed to the early prepupal stage compared to the control. The early molting at these groups was also supported by the higher weight recorded in all subsequent development stages, namely, in the prepupal, pupal and imago phases (Table 1).

In the prepupal stage, the weight of the individuals fed with vegetable oils was higher (*p* < 0.05) than in the control. The heaviest weight was observed in the soybean oil treatment, followed by the rapeseed oil treatment (Table 1).

In the pupal stage, the higher weight (*p* < 0.05) of the individuals from the diets with soybean and rapeseed oil was maintained, but the pupae weight from the diets with sunflower and hempseed oil was not significantly different (*p* > 0.05) compared to that of the control (Table 1).

**Figure 1:**
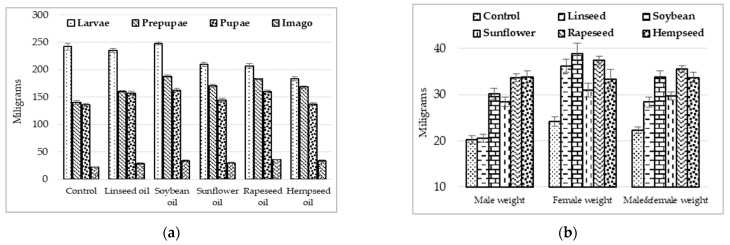
The effects of using different vegetable oils in the larval rearing substrate on the body weight evolution in different life stages of *Hermetia illucens*: larvae to imago stages (**a**); imago stages (**b**).

The weight of flies (males and females) from the diets with vegetable oils was clearly higher (*p* < 0.05) than that of the control (Table 1). Of these, the highest fly weight was found for the treatments with rapeseed, soybean, and hempseed oils (Figure 1). A particular situation was observed for linseed oil, where the male weights were lower and close to the control (*p* > 0.05) (Table 1), but the female weights were close to the groups with the best performance (soybean and rapeseed oil) (*p* > 0.05) (Figure 1).

The body weight loss from the larval stage to the prepupa stage was lower for the individuals provided with diets with hempseed and rapeseed oil, and higher for the individuals in the control and those in the linseed and soybean oil treatments (Figure 1).

The body weight loss from prepupa to pupa stages was the lowest in control and in the linseed oil treatment and more accentuated for the others. For the diets with sunflower oil, hempseed oil, and control, the body weight loss from pupa to imago was lower compared to the other groups (Figure 1).

### 3.2. Reproductive Performances of H. illucens Individuals

Utilization of different vegetable oils in the feeding of *H. illucens* larvae did not influence the adult reproductive performances, namely, the egg weight (*p* > 0.05) and the egg number in the clutch (*p* > 0.05) (Table 2).

However, the best egg mass production was obtained in the control and in the group with linseed oil, while in the rapeseed oil group, the eggs production was the lowest, which was also supported by the lowest (*p* < 0.001) mean egg weight recorded here (Table 2).

### 3.3. Larvae Crude Fat Content and Fatty Acid Profile

**Figure 2:**
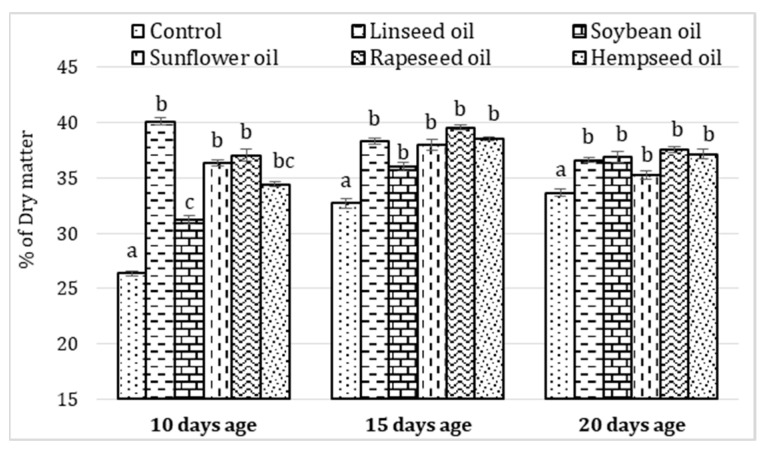
The effects of vegetable oil addition in the *Hermetia illucens* larvae diets on the crude fat content (% of DM) at three age categories (Mean ± SE). Different letters (a–c) above means indicate significant differences (*p* < 0.05).

The use of vegetable oils in feeding *H. illucens* larvae led to the obtaining of larvae with a higher crude fat content (*p* < 0.05) at each evaluated age (Figure 2). The larvae fat content increased until the age of 15 days, after which a decrease was observed. However, all the larvae that benefited from oil in the diet had a higher crude fat content (*p* < 0.05) (Figure 2).

At the larvae age of 10 days, the highest crude fat content level was observed for individuals with the linseed oil diet, and at the age of 15 and 20 days, the highest fat content was found in the larvae from rapeseed and hemp oil diets, followed closely by the larvae from the linseed oil diet (Figure 2).

Including the vegetable oils in larvae feeding strongly influenced the fatty acid profile of larvae fats at each evaluated age (Table 3, Table 4 and Table 5).

Fats from 10-day-old *H. illucens* larvae were characterized by the major presence of lauric acid (18.67–44.75% of FAME) followed by palmitic acid (18.72–24.10% of FAME) (Table 3). The inclusion of vegetable oils rich in unsaturated fatty acids (UFA) in the diet led to a decrease (*p* < 0.001) in the proportion of saturated fatty acids (SFA) and to an increase (*p* < 0.001) in the UFA concentration in fats, compared to the control (Table 3). In the case of the experimental diets, the highest level in SFA and the lowest in UFA were recorded in the larvae from the sunflower oil diet, followed by those from the soybean oil diet. In addition, when the vegetable oils were added to the larval feed, higher lipid quality indices of fats (*p* < 0.001) were obtained (Table 3). The improvement of fat lipid quality indices consisted of increasing the concentration of n–3 PUFA fatty acids in the larvae, which significantly increased the degree of fat polyunsaturation (*p* < 0.001) and decreased the value of the n–6/n–3 ratio (Table 3). The larvae fats from diets containing oils of linseed, hemp, and rapeseed had higher contents (*p* < 0.05) of linoleic acid (C18: 2 n–6), γ–linolenic acid (C18: 3 n–6), and α–linolenic acid (C18: 3 n–3) compared to the others (Table 3). The use of linseed oil led to the highest concentration of UFA (48.74% of FAME) in the larvae (*p* < 0.05). Therefore, the fats had a significantly higher polyunsaturation index (PI = 41.44%), which led to a decrease (*p* < 0.05) in the value of the atherogenic (AI = 1.22%) and thrombogenic index (TI = 0.48%) compared to the other groups. The results obtained in the case of linseed oil were followed by those obtained for hemp and rapeseed oil.

At the larvae age of 15 days, the predominant fatty acids in fats were lauric (18.86–41.37% of FAME) and palmitic (17.07–24.89% of FAME) in all groups (Table 4). Although SFA predominated in all groups, their proportion decreased (*p* < 0.001) (84.82–50.50% of FAME) when oils were included in the larvae diets, which led to the increase (*p* < 0.001) of UFA concentration (12.65–47.13% of FAME) (Table 4). Among the monounsaturated fatty acids (MUFAs), the oleic fatty acid predominated (C18: 1n–9), with the highest level (*p* < 0.05) in the larvae from the rapeseed oil diet (10.05% of FAME). The α-linolenic acid (C18: 3n–3) predominated (*p* < 0.05) among the polyunsaturated fatty acids (PUFAs) in the larvae that received linseed oil in the diet. Linoleic fatty acid (C18: 2n–6) predominated in larvae that received soybean oil in the diet; and γ–linolenic acid (C18: 3n–6) predominated in the larvae that received sunflower, rapeseed, or hempseed oil in the diet.

The higher content of PUFA in the larvae fats contributed to the improvement (*p* < 0.001) of lipid quality indices, with an increase (*p* < 0.001) of the omega–3 fatty acid series compared to the control. At the age of 15 days of larvae, the use of linseed oil in feed led to the highest (*p* < 0.05) concentration of UFA (47.13% of FAME), which positively influenced the fat quality indices, being higher than the others. For example, the larvae fats provided by the linseed oil diet had the lowest (*p* < 0.05) values of the atherogenic and thrombogenic index (Table 4).

In the fats of 20-day-old *H. illucens* larvae, SFAs predominated (52.90–85.86% of FAME) to the detriment of UFAs (11.24–44.90% of FAME). The MUFAs predominated in the larvae of the control and in those of the sunflower oil diet, while the PUFAs predominated in the larvae fed with linseed, hemp, rapeseed, and soybean oil in diets (Table 5).

The effects of adding vegetable oils to the feeding of 20-day-old larvae consisted of increasing (*p* < 0.001) the degree of fat unsaturation, especially polyunsaturation, which contributed significantly to improving the larvae fat quality by increasing (*p* < 0.001) the n–3 series fatty acids. Therefore, the larvae in the diet with linseed oil, followed by hemp and rapeseed oils, had the highest values of the polyunsaturation index (*p* < 0.05), and the lowest (*p* < 0.05) values of the atherogenic and thrombogenic indices (*p* < 0.05) (Table 5).

### 3.4. Evolution of Fatty Acid Content Depending on Larvae Age

**Figure 3:**
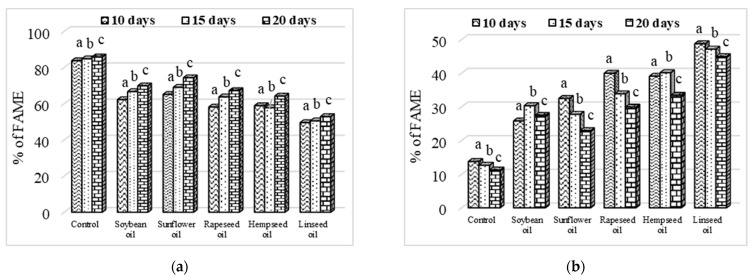
Evolution of saturated fatty acids (**a**) and unsaturated fatty acids (**b**) according to larvae age. Different letters (a–c) above means indicate intragroup significant differences at *p* < 0.05.

The evolution of the FA concentration according to the larvae age (10, 15, and 20 days) revealed that at the age of 10 days, the SFA had the lowest level, except in the larvae fed with hempseed oil; UFAs had the highest level, except for the larvae provided from diets with soybean oil and hempseed oil (Figure 3). According to Figure 3, the SFA concentration in the larvae fats increased (*p* < 0.001) with age development; therefore, at the age of 20 days, the SFA was found in the highest concentration in all larvae. Additionally, the level of UFA decreased (*p* < 0.001) as the larvae grew older, so at 20 days, the UFAs had the lowest levels (Figure 3).

**Figure 4:**
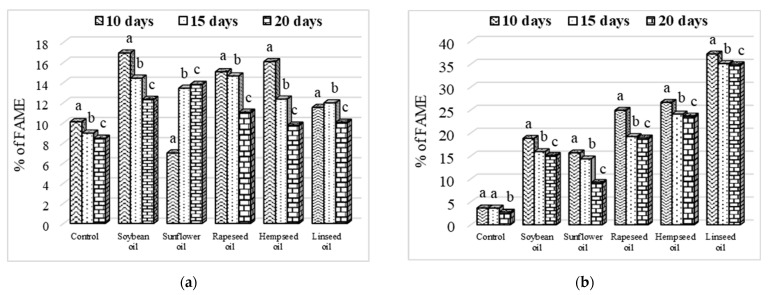
Evolution of monounsaturated (**a**) and polyunsaturated (**b**) fatty acids according to larvae age. Different letters (a–c) above means indicate intragroup significant differences at *p* < 0.05.

The level of MUFAs and PUFAs decreased with the larvae age development; therefore, at the age of 20 days, the larvae had the lowest levels of MUFAs and PUFAs (Figure 4).

However, compared to the control, the effects of improving the quality of *H. illucens* larvae fats through the use of vegetable oils in diets were not influenced by the larvae age, because even if PUFAs decreased at 20 days old of larvae, their levels still remained significantly higher than those of the larvae in the control.

## 4. Discussion

### 4.1. Performance Response

The significantly higher weight recorded by *Hermetia illucens* larvae from diets with added oils may be due to the higher energy level available. It is well known that *H. illucens* larvae respond positively by accumulating body weight mass when the rearing substrate has a higher energy level [27,48,49]. Li et al. [35] increased the supplementation of larval feed with vegetable oil from 5% to 10% and observed an increased in the larvae weight. The larvae phase is the only biological stage in which the species feeds; therefore, it accumulates body mass to support the reproductive process and the reserves serving as energy for all subsequent stages [16,37,49]. Therefore, in all diets, the larvae weight is higher at the age of 15 days than at 10 days (Table 1). However, at 20 days, the weights of individuals from diets with oils were lower than those of the control. This could have occurred due to the accelerated larvae growth (as an effect of a higher energy level of diets), which generated a larval stage shortening effect (at 19–20 days) at the same time as the weight reduction due to the molting, the individuals being already in the prepupae early stage. At this age, larvae from the control had not yet molted, resulting in a higher weight. In this way, Oonincx et al. [24] obtained a larval stage duration of 17 days when the rearing diet was enriched with 4% flaxseed oil, and the larvae weight reported by the authors is lower compared to our results (145 ± 11.4 mg vs. 234 ± 3.91 mg) possibly due to the smaller amount of flaxseed oil used. Additionally, our observation can be supported by the findings of Li et al. [35], who obtained prepupae at 19 days after hatching the larvae from a substrate enriched with 10% flaxseed oil and 10% soybean oil. Another hypothesis may be that the role and functions of certain fatty acids from diets in larvae differ, as suggested by Hoc et al. [50], since the protein and energy levels of the feed did not vary between the diets with oils, but significant differences were obtained mainly between the diets with flaxseed oil and soybean oil on one hand, and sunflower oil and rapeseed oil on the other hand. Additionally, Li et al. [35] showed that the level of palmitic acid (C16:0) in the diet was the major factor influencing the final larvae weight, the authors attributing the significant variation of the weight of larvae reared on different diets containing flaxseed oil, peanut, coconut, soy, lard, and fish to palmitic acid. According to the authors, the higher larvae weight was obtained when the diet had a higher content of palmitic acid, results that are consistent with ours, because the soybean oil diet had the larvae with the highest final weight, but also with the highest level of palmitic acid. The role of fatty acids such as C18:0, C18:3 n-3, and C18:2 n-6 is well known in other insect species during the larval development, pre-pupation rate, pupation rate, and emergence rate [51,52,53,54]. The higher larvae weight from the oil diets led to the obtaining of prepupae, pupae, and adults with higher weights than the control (*p* < 0.05). Hoc et al. [34] reported a prepupae weight of 110.79–204.04 mg when *Hermetia illucens* larvae were reared on substrates containing different levels of flax cake, and 126.56–206.69 mg for rape cake, ranges that are consistent with our results. The prepupae weight obtained by us in the case of flaxseed oil was 160.27 mg, and for rapeseed oil it was 183.07 mg. Li et al. [35] also reported a final larvae body weight of 100.0 mg obtained when adding 10% soybean oil in the feed, which is lower compared to the larvae weight obtained by us in the case of the soybean oil diet (247.25 mg). According to our results, the weight of 20-day-old larvae from the soybean oil diet was higher than that of larvae from the flaxseed oil diet (247.25 mg vs. 234.83 mg), without being statistically significant; however, this aspect is consistent with the findings reported by Li et al. [35].

Enrichment of the larval rearing substrate with vegetable oils did not influence (*p* > 0.05) the egg clutch weight and the number of eggs in a clutch. The obtained clutch weight was close to the values reported in other studies, such as 14.5–15.9 mg [16] or 10.53–12.23 mg [55]. The total number of eggs was comparable to that reported by Tomberlin et al. [16], namely, 603–689 eggs; by Barros et al. [23] (620–700 eggs); and by Bertinetti et al. [55] (412–1060) eggs.

### 4.2. Fats and Fatty Acid Profile of Larvae

The larvae crude fat content increased when vegetable oils were added in the rearing substrate, as a result of the diet energy level increase. This aspect is consistent with the results obtained by Li et al. [35], who reported an increase in the crude fat of *H. illucens* larvae as well as a decrease in the crude protein and ash content when the inclusion level of dietary oils increased from 5 to 10%. For *H. illucens*, the larvae crude fat level is increased when the diet energy level is higher and decreased if the protein level is higher [31,56]. Accumulating body fat in the larval stage occurs because the fat reserves are vital for supporting the reproductive process [16,49,57]. The crude fat level in larvae (30–40%) found by us is higher than that obtained by Li et al. [35], who reported a content of 20–25% in larvae reared on diets supplemented with 10% of flaxseed oil and soybean oil, respectively. The findings of Li et al. [35], namely, that a higher crude fat level was obtained when the diet was rich in SFA because *H. illucens* larvae can synthesize acetyl-CoA through saturated fatty acids C12:0, C14:0, C16:0, C18:0 and form triglyceride [58], is not consistent with our findings. We obtained a higher fat level in the larvae from the diets to which oils were added, so with a higher energy level, but also a lower SFA level, which proves the fact that the diet energy value could be the major factor that determines the fat level in larvae.

In our research we found that the fats of *H. illucens* larvae were characterized by the high presence of SFA, especially lauric, palmitic, and myristic acid, even if the diets were supplemented with oils rich in unsaturated fatty acids. Previously, Hoc et al. [34] showed that the fatty acid profile is dominated by SFA in the prepupae lipids, especially lauric (40.41–55.59% of FAME), even if the larvae were reared on flax and rapeseed cakes. According to the conclusions of Li et al. [35], the high level of lauric and myristic acid in the larvae fats may be due to the larvae synthesizing these fatty acids even if they are not found in the diet. It is also well known that *H. illucens* larvae have the ability to synthesize lauric acid, which is the most abundant fatty acid [21,34]. This appears to be due to the unique ability of *H. illucens* to de novo synthesize lauric acid without it being present in the diet [24,34,59]. It can be synthesized from sugars [32]. In our research, this aspect was confirmed by the high level of lauric acid in larvae regardless of diet (18.67–44.75% of FAME) or age.

The role and synthesis of fatty acids in *H. illucens* larvae are not clear and, therefore, require further study. Ewald et al. [21] found a positive correlation between the larvae weight and the lauric fatty acid level (R^2^ = 0.8), as well as total SFA (R^2^ = 0.7) in larvae, and a negative correlation with MUFA (R^2^ = 0.5) and PUFA (R^2^ = 0.7). Li et al. [35] suggested that eicosapentaenoic acid (C20:5 n-3) and docosahexaenoic acid (22: 6 n-3) may have implications in larval growth. In our research, the larvae that received oils rich in PUFA in diets had a higher weight compared to larvae from the control, and implicitly higher amounts of EPA acid. The content of omega-3 PUFAs, especially α-linolenic, is high in flaxseed, hempseed and rapeseed oil [1,60], which led to higher concentrations of omega-3 FA in larvae, without affecting (*p* > 0.05) the development of larvae, prepupae, pupae, adults, and reproductive parameters. Similar findings on the optimal development of *H. illucens* as a result of n-3 PUFA enrichment of larval fat through the diet were reported by Oonincx et al. [24] when flaxseed oil was used.

The effects of adding oils rich in UFA are represented by a clear modification of the profile and ratio of FA in larvae, by the decreasing proportion of SFA in parallel with the increasing UFA proportion, especially PUFA, according to the oil used. The modulation of the larvae fatty acid profile through the diet has also been previously demonstrated by Hoc et al. [34], according to whom the fatty acid profile of prepupae differs depending on the flaxseed or rapeseed meal used in the larvae diet. Simultaneously, the authors reported that the progressive addition of flax and rapeseed cakes in the larval diet (10–100%) led to the progressive decrease of SFA in the obtained prepupae, along with the progressive increase of MUFA and PUFA, showing the strong influence of rearing substrate quality, an aspect that we also observed.

Among the PUFA acids of the larvae fats, α-linolenic fatty acids (ALAs) were predominant, followed by γ–linolenic acid, when flaxseed oil was used, and linoleic and γ-linolenic fatty acids, when rapeseed or hempseed oils were used. Similar findings were reported by Li et al. [35], who obtained a predominance of α–linolenic acid in the larvae fat fed with a diet containing 10% flaxseed oil, and by Hoc et al. [34] for larvae reared on flax cakes. The use of flax cakes up to 100% of the diet led to an ALA level of 15.2% of FAME, being slightly higher than in our findings (10.05–14.85% of FAME). Thus, we can appreciate that enriching diets with oils could be a more effective solution. Among MUFAs, oleic fatty acid predominates in all larvae fats, being the unsaturated fatty acid with the highest concentration in the larvae that received soybean and rapeseed oil in the diet (Table 3, Table 4 and Table 5). Similarly, Li et al. [35] observed oleic acid as the predominant monounsaturated fatty acid in larval fat, being most abundant of all UFA in larvae that received 10% soybean oil in the diet. Additionally, Hoc et al. [34] reported oleic acid as predominant in the fat of prepupae provided from larvae reared on flax and rapeseed cakes. According to the authors, the rapeseed cakes are rich in oleic fatty acid, which was found consistently in the fatty acid profile of prepupae, aspects we also found in the larvae that received rapeseed oil.

The obtained results prove that *H. illucens* larvae do not have the ability to synthesize UFAs, their level and structure being dependent on their concentration in the rearing diet. Research by Giannetto et al. [28] showed that the degree of enrichment of larval fats in PUFA generally follows the level of PUFA in the rearing diet, which shows that the species is not able for de novo synthesis of PUFA. Oonincx et al. [36] found a correlation between the addition of flaxseed oil and the ALA level in larvae, obtaining for each percentage of flaxseed oil added in the diet of larvae a 2.3–2.7% increase of ALA, being higher than our findings (1.05–1.45%). The EPA and DHA fatty acids were not found in prepupae fat when they had not existed in the larvae diet [21,23,36]. Recently, Li et al. [35] have observed that the levels of oleic (C18:1 n-9), linoleic (C18:2 n-6), and α–linolenic (C18:3 n-3) acids in larvae are positively influenced by the level from the larvae diet.

The profile and fatty acid ratio have strong variations, depending on the diet and larvae age. Thus, Giannetto et al. [28] showed a variation in the concentration of fatty acids influenced by the development stage of *H. illucens*, having a level in ALA twice lower in the prepupae stage than in the last instar of larvae. The authors claimed that the differences could be due to the variation of the enzymatic activity involved in the fatty acid metabolism. These statements support our findings; we obtained a higher concentration of ALA in 10-day-old larvae than in 20-day-old larvae. Thus, the potential of modelling the larvae fatty acid profile even by age is noted.

The enrichment in PUFA of the *H. illucens* larvae fats further used in fish feed leads to a food source for human consumption with a high level of omega–3, useful for human cardiovascular disease prevention [5,17]. This highlights the importance of monitoring omega–3 fatty acids in livestock production, which lead to higher quality indices of fats beneficial to human health. In the present research, the increase in the proportion of omega-3 fatty acids in larvae had a positive effect, which led to a decrease in the value of the n-6/n-3 ratio. Similar positive effects have been previously reported by Hoc et al. [34] for prepupae fat from larvae reared on rapeseed and flax cakes. According to Oonincx et al. [24], the inclusion of 4% flaxseed oil in the larvae diet led to an increase in the omega-3 level, which favored a strong decrease in the n–6/n–3 ratio from 18–36 to 0.8–2.4. A similar effect was obtained by us, where the ratio of n–6/n–3 decreased from 8.22 to 1.39–2.24 when 10% flaxseed oil was added compared to the control. A ratio of less than 5 is considered optimal for human health [5,61]. In the larvae from diets with flaxseed, hempseed, and rapeseed oil, the ratio of n–6/n–3 was less than 5, regardless of age, but the higher values were obtained at the 10 days age of the larvae.

Several researches aimed to enrich the fatty acid profile of the larvae with omega–3 by using fish offal or fishmeal, or vegetable by-products (seaweed, flaxseeds), and obtained an increase up to 2.99–16.5% of the omega–3 PUFA level [21,23,32,44,56]. Oonincx et al. [24] showed that the fatty acid profile differs depending on the species of insect used (*Acheta domesticus*, *Alphitobius diaperinus*, and *Hermetia illucens*); even though insects were reared on similar diets, they responded similarly to the addition of flaxseed oil in the diet (1–4%). Finke et al. [62] enhanced the fatty acid composition of crickets, mealworms, superworms, and waxworms to make them a better diet for insectivores.

The aquaculture industry is highly dependent on fishmeal and fish oil; therefore, *H. illucens* meal can be a sustainable alternative source of proteins and fats with improved nutritional quality. This is feasible, because, compared to the research conducted by Li et al. [35], who used fish oil in larvae feed, we obtained a considerably lower level of SFA (61.1 vs. 49.5–52.9% of FAME) when flaxseed oil was used in the diet, and up to twice as high in PUFAs in the case of flaxseed, hemp, and rapeseed oil (24.4 vs. 18.9–37.2% of FAME). Therefore, these findings indicate the superiority of oil use in enrichment of the fatty acid profile of *H. illucens* larvae compared to fish oil. When the *H. illucens* larvae meal with an unbalanced fatty acid profile was used in fish feed, the content of ALA, EPA, and DHA decreased in fish fillets [63]. Replacing the fish oil in the *Cyprinus carpio* feed with *H. illucens* larvae oil up to 100% led to a significant increase in the proportion of SFA together with the decrease (*p* < 0.05) in the proportion of MUFA and n–6 PUFA in the muscle [64]. Omega–3 FAs, especially EPA and DHA, are essential for optimal fish growth and reproduction [65] and are also associated with multiple health benefits for human consumers [66]. Therefore, low concentrations of n–3 series FAs in fish fed with *H. illucens* larvae may be a problem for both the breeder and human consumers [21]. ALA can be converted to EPA and DHA by several enzymatic steps, and the same enzymes can elongate in linoleic acid [67]. Therefore, if insects are to be part of a sustainable diet that meets the nutrient requirements of animal species, it would be advantageous to contain relatively high proportions of n-3 PUFAs, which would result in more favorable n–6/n–3 ratios [36].

Integrated into a breeding technology, enriching the fatty acid profile of *H. illucens* larvae with oils can solve the problems of limiting its use in aquaculture due to the unbalanced natural profile. In addition, future research could see if the larvae accumulate fatty acids from the enriched diet in a shorter time, so that in 1–2 days this can already be achieved, possibly being more cost-effective than a long-term supply.

## 5. Conclusions

The use of vegetable oils in the *Hermetia illucens* larvae rearing diets positively influenced the weight of larvae, prepupae, pupae, and adults without affecting reproductive performances (clutch weight and number of eggs in a clutch). The addition of oils in larvae diets led to an increase (*p* < 0.05) in the crude fat content and to modifications in the fatty acid profile of fats. The use of flaxseed oil favored the obtaining of the best fatty acid profile, regardless of the age of larvae, followed by hemp and rapeseed oil. By enriching fats with UFA, especially polyunsaturated fats, the larvae have higher concentrations of omega-3 fatty acids (up to 15.55% of FAME), favoring a high degree of fat polyunsaturation (PI = 17.76–41.44) and low values of atherogenic (AI = 3.22–1.22) and thrombogenic (TI = 1.43–0.48) indices. The larvae have the highest concentrations of UFA at 10 days age, decreasing with age (*p* < 0.05). Therefore, it is recommended to use 10 to 15-day-old larvae as sources for feed. Enrichment of larvae-rearing diets with sources rich in UFA, such as oils, can be considered the key when modelling the fatty acid profile of *H. illucens* larvae, given that they are not capable of de novo synthesis of UFA. Thus, the larvae enriched in PUFA are an excellent source of nutrients for fish feeding and other species with economic interest, which can contribute substantially to obtaining high quality animal products for human consumption, successfully contributing to human health.

## Figures and Tables

**Table 1:** The effects of vegetable oil addition on the larvae diets on the evolution of body weight in the developmental stages of *Hermetia illucens*.

Specification		Control	Experimental Treatments (Mean ± SE)					*p*-Value
			Linseed Oil	Soybean Oil	Sunflower Oil	Rapeseed Oil	Hempseed Oil	
Larvae weight	10 days age	142.47 ± 3.18 ^a^	162.26 ± 2.62 ^bc^	165.64 ± 2.52 ^b^	169.45 ± 2.47 ^b^	152.25 ± 1.73 ^ac^	183.43 ± 4.17 ^d^	0.001
	15 days age	239.74 ± 2.75 ^a^	282.77 ± 2.90 ^b^	279.92 ± 2.84 ^b^	261.32 ± 2.86 ^c^	262.75 ± 2.89 ^c^	246.69 ± 2.86 ^a^	0.001
	20 days age	241.80 ± 6.18 ^a^	234.83 ± 3.91 ^a^	247.25 ± 2.65 ^a^	209.27 ± 3.70 ^b^	206.82 ± 4.50 ^b^	183.55 ± 3.62 ^c^	0.001
Prepupae weight		140.18 ± 3.56 ^a^	160.27 ± 1.66 ^b^	187.76 ± 2.29 ^d^	170.39 ± 2.54 ^bc^	183.07 ± 1.07 ^dc^	168.20 ± 2.34 ^b^	0.001
Pupae weight		135.48 ± 3.52 ^a^	157.27 ± 3.64 ^bc^	162.66 ± 3.56 ^b^	144.72 ± 3.35 ^ac^	160.17 ± 2.85 ^b^	136.99 ± 2.82 ^a^	0.001
Male weight		20.28 ± 0.88 ^a^	20.57 ± 0.83 ^a^	30.16 ± 1.15 ^bc^	28.49 ± 1.03 ^c^	33.70 ± 0.88 ^b^	33.86 ± 1.30 ^d^	0.001
Female weight		24.22 ± 1.05 ^a^	36.21 ± 1.43 ^b^	38.79 ± 2.40 ^b^	30.93 ± 1.47 ^c^	37.41 ± 0.96 ^b^	33.36 ± 2.15 ^bc^	0.001
Male and female weight		22.25 ± 0.71 ^a^	28.39 ± 1.14 ^b^	33.86 ± 1.34 ^c^	29.71 ± 0.90 ^b^	35.55 ± 0.67 ^c^	33.69 ± 1.11 ^c^	0.001

SE: standard error; ^a–d^: values within a column followed by the different superscript letter are significantly different (*p* < 0.05).

**Table 2:** The effects of vegetable oil addition in the larvae rearing diets on the reproductive performances of *Hermetia illucens*.

Parameters	Control	Experimental Treatments (Mean ± SE)					*p*-Value
		Linseed Oil	Soybean Oil	Sunflower Oil	Rapeseed Oil	Hempseed Oil	
Clutch weight (mg)	19.97 ± 1.49 ^a^	17.74 ± 2.25 ^a^	16.70 ± 0.89 ^a^	17.97 ± 1.28 ^a^	15.57 ± 1.60 ^a^	17.75 ± 1.46 ^a^	0.361
Eggs number/clutch	852.30 ± 45.91 ^a^	771.50 ± 83.48 ^a^	794.36 ± 47.88 ^a^	748.00 ± 49.62 ^a^	850.67 ± 96.14 ^a^	799.24 ± 76.11 ^a^	0.672
Egg weight (mg)	0.0234 ± 0.01 ^a^	0.0226 ± 0.01 ^a^	0.0213 ± 0.01 ^ab^	0.0240 ± 0.01 ^a^	0.0186 ± 0.01 ^b^	0.0223 ± 0.01 ^a^	0.001
Total clutches (n)	41	48	42	32	31	28	-

SE: standard error; ^a,b^: values within a column followed by the different superscript letter are significantly different (*p* < 0.05).

**Table 3:** The influence of vegetable oil inclusion in the diets of 10-day-old larvae on the fatty acid profile of fats (% of FAME).

Fatty Acids	Control	Experimental Treatments (Mean ± SE)					*p*-Value
		Linseed Oil	Soybean Oil	Sunflower Oil	Rapeseed Oil	Hempseed Oil	
Lauric acid (C12:0)	44.75 ± 0.07 ^a^	18.67 ± 0.05 ^b^	21.32 ± 0.08 ^c^	28.74 ± 0.03 ^d^	23.95 ± 0.06 ^e^	25.25 ± 0.06 ^f^	0.001
Miristic acid (C14:0)	7.39 ± 0.07 ^a^	5.64 ± 0.07 ^b^	7.52 ± 0.09 ^a^	6.78 ± 0.06 ^c^	5.87 ± 0.06 ^d^	7.21 ± 0.05 ^a^	0.001
Palmitic acid (C16:0)	23.93 ± 0.03 ^a^	18.72 ± 0.07 ^b^	24.10 ± 0.05 ^c^	21.07 ± 0.03 ^d^	18.66 ± 0.07 ^b^	20.16 ± 0.06 ^e^	0.001
Stearic acid (C18:0)	7.69 ± 0.07 ^a^	6.36 ± 0.07 ^b^	9.34 ± 0.09 ^c^	8.45 ± 0.07 ^d^	9.66 ± 0.07 ^e^	6.41 ± 0.05 ^b^	0.001
Palmitoleic acid (C16:1)	4.80 ± 0.06 ^a^	3.25 ± 0.07 ^b^	5.53 ± 0.06 ^c^	4.25 ± 0.07 ^d^	4.41 ± 0.05 ^d^	4.45 ± 0.08 ^d^	0.001
Oleic acid (C18:1n-9)	5.28 ± 0.07 ^a^	8.29 ± 0.09 ^b^	11.45 ± 0.07 ^c^	12.58 ± 0.03 ^d^	10.66 ± 0.07 ^e^	7.92 ± 0.01 ^f^	0.001
Linoleic acid (C18:2n-6) (LA)	2.74 ± 0.04 ^a^	11.74 ± 0.07 ^b^	9.03 ± 0.02 ^c^	8.93 ± 0.02 ^c^	10.25 ± 0.07 ^d^	12.54 ± 0.09 ^e^	0.001
γ–linolenic acid (C18:3n-6)	0.53 ± 0.05 ^a^	9.89 ± 0.03 ^b^	4.78 ± 0.06 ^c^	4.76 ± 0.06 ^c^	8.04 ± 0.02 ^d^	8.40 ± 0.07 ^e^	0.001
α–linolenic acid (C18:3n-3) (ALA)	0.7 ± 0.06 ^a^	14.85 ± 0.04 ^b^	4.55 ± 0.07 ^c^	1.74 ± 0.07 ^d^	6.24 ± 0.07 ^e^	5.24 ± 0.07 ^f^	0.001
Eicosenoic acid (C20:5n-3)	0.13 ± 0.02 ^a^	0.71 ± 0.06 ^b^	0.45 ± 0.02 ^c^	0.21 ± 0.02 ^ac^	0.33 ± 0.03 ^ac^	0.45 ± 0.02 ^bc^	0.008
Others fatty acids	2.06 ± 0.02 ^a^	1.88 ± 0.04 a	1.93 ± 0.02 ^a^	2.49 ± 0.05 ^a^	1.93 ± 0.03 ^a^	1.97 ± 0.04 ^a^	0.078
SFA	83.76 ± 0.07 ^a^	49.56 ± 0.11 ^b^	62.24 ± 0.07 ^c^	65.13 ± 0.09 ^d^	58.18 ± 0.06 ^e^	58.19 ± 0.04 ^f^	0.001
UFA	13.76 ± 0.07 ^a^	48.74 ± 0.06 ^b^	35.81 ± 0.05 ^c^	32.50 ± 0.10 ^d^	39.96 ± 0.02 ^e^	39.09 ± 0.02 ^f^	0.001
MUFA	10.10 ± 0.03 ^a^	11.52 ± 0.07 ^b^	16.92 ± 0.05 ^c^	16.83 ± 0.04 ^c^	15.06 ± 0.01 ^d^	12.35 ± 0.07 ^e^	0.001
PUFA	3.66 ± 0.06 ^a^	37.22 ± 0.07 ^b^	18.84 ± 0.03 ^c^	15.67 ± 0.07 ^d^	24.95 ± 0.02 ^e^	26.67 ± 0.07 ^f^	0.001
n–3	0.38 ± 0.05 ^a^	15.55 ± 0.07 ^b^	5.04 ± 0.02 ^c^	1.91 ± 0.03 ^d^	6.67 ± 0.07 ^e^	5.75 ± 0.08 ^f^	0.001
n–6	3.27 ± 0.07 ^a^	21.66 ± 0.07 ^b^	13.87 ± 0.05 ^c^	13.76 ± 0.07 ^c^	18.24 ± 0.05 ^d^	20.88 ± 0.09 ^e^	0.001
UFA/SFA	0.16 ± 0.04 ^a^	0.97 ± 0.05 ^b^	0.56 ± 0.05 ^c^	0.50 ± 0.03 ^c^	0.67 ± 0.05 ^c^	0.67 ± 0.06 ^c^	0.001
PUFA/SFA	0.04 ± 0.01 ^a^	0.77 ± 0.01 ^b^	0.31 ± 0.02 ^c^	0.24 ± 0.04 ^ac^	0.43 ± 0.03 ^c^	0.44 ± 0.03 ^c^	0.001
MUFA/SFA	0.12 ± 0.01 ^a^	0.25 ± 0.02 ^b^	0.26 ± 0.04 ^b^	0.27 ± 0.01 ^b^	0.26 ± 0.01 ^b^	0.21 ± 0.02 ^ab^	0.001
n–6/n–3	8.22 ± 0.09 ^a^	1.39 ± 0.04 ^b^	2.77 ± 0.07 ^c^	7.05 ± 0.12 ^c^	2.77 ± 0.04 ^d^	3.66 ± 0.08 ^e^	0.001
Polyunsaturated index (PI)	3.24 ± 0.04 ^a^	41.44 ± 0.07 ^b^	18.15 ± 0.11 ^c^	12.43 ± 0.07 ^d^	22.77 ± 0.09 ^e^	22.95 ± 0.02 ^e^	0.001
Atherogenic index (AI)	7.15 ± 0.03 ^a^	1.22 ± 0.07 ^b^	2.13 ± 0.04 ^ce^	2.37 ± 0.06 ^c^	1.66 ± 0.07 ^d^	1.91 ± 0.04 ^e^	0.001
Thrombogenic index (TI)	4.86 ± 0.02 ^a^	0.48 ± 0.01 ^b^	1.34 ± 0.02 ^c^	1.75 ± 0.02 ^d^	0.94 ± 0.01 ^e^	0.98 ± 0.01 ^e^	0.001

SE: standard error; ^a–f^: values within a column followed by the different superscript letter are significantly different (*p* < 0.05); SFA: saturated fatty acids, MUFA: monounsaturated fatty acids; PUFA: polyunsaturated fatty acids; UFA: unsaturated fatty acids; n-3: omega-3 fatty acids; n-6: omega-6 fatty acids.

**Table 4:** The influence of vegetable oil inclusion in the diets of 15-day-old larvae on the fatty acid profile of fats (% of FAME).

Fatty Acids	Control	Experimental Treatments (Mean ± SE)					*p*-Value
		Linseed Oil	Soybean Oil	Sunflower Oil	Rapeseed Oil	Hempseed Oil	
Lauric acid (C12:0)	41.37 ± 0.07 ^a^	18.86 ± 0.06 ^b^	30.79 ± 0.08 ^c^	34.94 ± 0.02 ^c^	28.73 ± 0.09 ^e^	23.50 ± 0.07 ^f^	0.001
Miristic acid (C14:0)	14.07 ± 0.03 ^a^	8.34 ± 0.05 ^b^	8.51 ± 0.06 ^b^	7.22 ± 0.07 ^c^	4.77 ± 0.09 ^d^	10.02 ± 0.04 ^e^	0.001
Palmitic acid (C16:0)	24.89 ± 0.04 ^a^	17.07 ± 0.02 ^b^	22.34 ± 0.07 ^c^	22.67 ± 0.08 ^d^	22.13 ± 0.09 ^c^	20.33 ± 0.04 ^e^	0.001
Stearic acid (C18:0)	4.49 ± 0.07 ^a^	6.26 ± 0.09 ^b^	5.24 ± 0.11 ^c^	4.13 ± 0.05 ^d^	8.12 ± 0.09 ^e^	3.84 ± 0.04 ^f^	0.001
Palmitoleic acid (C16:1)	4.06 ± 0.03 ^a^	4.05 ± 0.02 ^b^	6.12 ± 0.04 ^c^	5.55 ± 0.07 ^d^	4.56 ± 0.07 ^e^	6.65 ± 0.08 ^f^	0.001
Oleic acid (C18:1n-9)	4.93 ± 0.03 ^a^	7.96 ± 0.05 ^b^	8.24 ± 0.07 ^c^	7.88 ± 0.03 ^b^	10.05 ± 0.05 ^d^	9.43 ± 0.07 ^e^	0.001
Linoleic acid (C18:2n-6) (LA)	1.34 ± 0.08 ^a^	9.43 ± 0.08 ^b^	7.95 ± 0.06 ^c^	3.76 ± 0.07 ^d^	6.16 ± 0.07 ^e^	8.46 ± 0.08 ^f^	0.001
γ–linolenic acid (C18:3n-6)	1.03 ± 0.02 ^a^	11.04 ± 0.04 ^b^	6.56 ± 0.07 ^c^	8.54 ± 0.09 ^d^	9.91 ± 0.05 ^e^	9.55 ± 0.08 ^f^	0.001
α–linolenic acid (C18:3n-3) (ALA)	1.04 ± 0.03 ^a^	13.94 ± 0.04 ^b^	0.77 ± 0.01 ^c^	1.87 ± 0.03 ^d^	2.72 ± 0.03 ^e^	5.65 ± 0.07 ^f^	0.001
Eicosenoic acid (C20:5n-3)	0.23 ± 0.02 ^a^	0.67 ± 0.02 ^b^	0.66 ± 0.02 ^c^	0.33 ± 0.02 ^ad^	0.52 ± 0.02 ^bcd^	0.47 ± 0.02 ^d^	0.008
Others fatty acids	2.55 ± 0.04 ^a^	2.42 ± 0.05 ^a^	2.82 ± 0.06 ^a^	3.11 ± 0.04 ^b^	2.33 ± 0.03 ^a^	2.10 ± 0.06 ^a^	0.048
SFA	84.82 ± 0.05 ^a^	50.50 ± 0.15 ^b^	66.69 ± 0.09 ^c^	69.04 ± 0.06 ^d^	63.77 ± 0.07 ^e^	57.76 ± 0.08 ^f^	0.001
UFA	12.65 ± 0.08 ^a^	47.13 ± 0.03 ^b^	30.33 ± 0.07 ^c^	27.78 ± 0.07 ^d^	33.85 ± 0.04 ^e^	40.13 ± 0.03 ^f^	0.001
MUFA	8.97 ± 0.04 ^a^	11.97 ± 0.03 ^b^	14.43 ± 0.07 ^c^	13.43 ± 0.07 ^d^	14.65 ± 0.05 ^c^	16.07 ± 0.04 ^e^	0.001
PUFA	3.66 ± 0.07 ^a^	35.13 ± 0.06 ^b^	15.94 ± 0.03 ^c^	14.35 ± 0.05 ^d^	19.22 ± 0.08 ^e^	24.12 ± 0.07 ^f^	0.001
n–3	1.25 ± 0.06 ^a^	14.64 ± 0.07 ^b^	1.44 ± 0.07 ^a^	2.15 ± 0.07 ^c^	3.19 ± 0.08 ^d^	6.12 ± 0.09 ^e^	0.001
n–6	2.35 ± 0.07 ^a^	20.45 ± 0.08 ^b^	14.54 ± 0.04 ^c^	12.23 ± 0.09 ^d^	16.03 ± 0.04 ^e^	17.96 ± 0.08 ^f^	0.001
UFA/SFA	0.15 ± 0.01 ^a^	0.92 ± 0.02 ^b^	0.45 ± 0.02 ^c^	0.41 ± 0.02 ^c^	0.53 ± 0.02 ^d^	0.69 ± 0.03 ^e^	0.001
PUFA/SFA	0.04 ± 0.01 ^a^	0.69 ± 0.01 ^b^	0.24 ± 0.02 ^ac^	0.26 ± 0.02 ^ac^	0.32 ± 0.02 ^c^	0.44 ± 0.03 ^c^	0.001
MUFA/SFA	0.13 ± 0.03 ^a^	0.24 ± 0.04 ^a^	0.22 ± 0.02 ^a^	0.16 ± 0.03 ^a^	0.22 ± 0.06 ^a^	0.24 ± 0.04 ^a^	0.772
n–6/n–3	1.87 ± 0.06 ^a^	1.43 ± 0.08 ^b^	10.22 ± 0.06 ^c^	5.76 ± 0.07 ^d^	5.06 ± 0.08 ^e^	2.94 ± 0.03 ^f^	0.001
Polyunsaturated index (PI)	3.45 ± 0.07 ^a^	37.34 ± 0.07 ^b^	9.5 ± 0.05 ^c^	7.35 ± 0.09 ^d^	11.54 ± 0.11 ^e^	19.76 ± 0.07 ^f^	0.001
Atherogenic index (AI)	9.76 ± 0.07 ^a^	1.45 ± 0.08 ^b^	2.86 ± 0.06 ^c^	3.11 ± 0.04 ^d^	2.06 ± 0.03 ^e^	2.08 ± 0.03 ^e^	0.001
Thrombogenic index (TI)	4.33 ± 0.07 ^a^	0.55 ± 0.04 ^b^	1.94 ± 0.07 ^c^	1.77 ± 0.08 ^c^	1.35 ± 0.08 ^d^	0.96 ± 0.02 ^e^	0.001

SE: standard error; ^a–f^: values within a column followed by the different superscript letter are significantly different (*p* < 0.05); SFA: saturated fatty acids; MUFA: monounsaturated fatty acids; PUFA: polyunsaturated fatty acids; UFA: unsaturated fatty acids; n-3: omega-3 fatty acids; n-6: omega-6 fatty acids.

**Table 5:** The influence of vegetable oil inclusion in the diets of 20-day-old larvae on the fatty acid profile of fats (% of FAME).

Fatty Acids	Control	Experimental Treatments (Mean ± SE)					*p*-Value
		Linseed Oil	Soybean Oil	Sunflower Oil	Rapeseed Oil	Hempseed Oil	
Lauric acid (C12:0)	36.33 ± 0.07 ^a^	18.87 ± 0.06 ^b^	28.89 ± 0.02 ^c^	36.76 ± 0.07 ^d^	27.57 ± 0.06 ^e^	25.44 ± 0.09 ^f^	0.001
Miristic acid (C14:0)	17.24 ± 0.07 ^a^	8.86 ± 0.06 ^b^	10.07 ± 0.02 ^c^	9.44 ± 0.07 ^d^	11.56 ± 0.06 ^e^	10.95 ± 0.09 ^f^	0.001
Palmitic acid (C16:0)	24.44 ± 0.05 ^a^	20.44 ± 0.06 ^b^	26.14 ± 0.08 ^c^	23.89 ± 0.04 ^d^	22.22 ± 0.08 ^e^	22.06 ± 0.04 ^e^	0.001
Stearic acid (C18:0)	7.92 ± 0.04 ^a^	4.82 ± 0.05 ^b^	4.76 ± 0.07 ^b^	4.35 ± 0.06 ^c^	5.91 ± 0.07 ^d^	5.78 ± 0.04 ^d^	0.001
Palmitoleic acid (C16:1)	3.14 ± 0.03 ^a^	2.92 ± 0.07 ^b^	4.58 ± 0.05 ^c^	5.14 ± 0.05 ^d^	4.21 ± 0.05 ^e^	4.07 ± 0.09 b ^e^	0.001
Oleic acid (C18:1n-9)	5.32 ± 0.07 ^a^	6.14 ± 0.04 ^b^	7.77 ± 0.09 ^c^	8.65 ± 0.09 ^d^	6.78 ± 0.04 ^e^	5.65 ± 0.08 ^f^	0.001
Linoleic acid (C18:2n-6) (LA)	1.50 ± 0.09 ^a^	14.22 ± 0.07 ^b^	7.77 ± 0.05 ^c^	1.43 ± 0.06 ^a^	7.86 ± 0.07 ^c^	9.32 ± 0.09 ^d^	0.001
γ–linolenic acid (C18:3n-6)	0.67 ± 0.07 ^a^	10.05 ± 0.02 ^b^	4.44 ± 0.07 ^c^	5.49 ± 0.05 ^d^	5.94 ± 0.02 ^e^	7.66 ± 0.07 ^f^	0.001
α–linolenic acid (C18:3n-3) (ALA)	0.44 ± 0.07 ^a^	10.13 ± 0.06 ^b^	2.77 ± 0.07 ^c^	2.15 ± 0.04 ^d^	4.92 ± 0.04 ^e^	6.45 ± 0.07 ^f^	0.001
Eicosenoic acid (C20:5n-3)	0.14 ± 0.06 ^a^	0.46 ± 0.07 ^b^	0.23 ± 0.05 ^a^	0.16 ± 0.08 ^a^	0.16 ± 0.02 ^a^	0.26 ± 0.02 ^ab^	0.008
Others fatty acids	2.85 ± 0.06 ^a^	2.13 ± 0.04 ^a^	2.70 ± 0.07 ^a^	2.66 ± 0.04 ^a^	2.69 ± 0.07 ^a^	2.30 ± 0.04 ^a^	0.529
SFA	85.86 ± 0.06 ^a^	52.90 ± 0.08 ^b^	69.87 ± 0.03 ^c^	74.34 ± 0.07 ^d^	67.24 ± 0.08 ^e^	64.23 ± 0.09 ^f^	0.001
UFA	11.23 ± 0.07 ^a^	44.92 ± 0.04 ^b^	27.44 ± 0.07 ^c^	22.94 ± 0.04 ^d^	29.94 ± 0.02 ^e^	33.44 ± 0.07 ^f^	0.001
MUFA	8.46 ± 0.06 ^a^	10.05 ± 0.02 ^b^	12.32 ± 0.08 ^c^	13.81 ± 0.12 ^d^	11.05 ± 0.17 ^e^	9.76 ± 0.09 ^f^	0.001
PUFA	2.76 ± 0.08 ^a^	34.88 ± 0.05 ^b^	15.13 ± 0.03 ^c^	9.22 ± 0.07 ^d^	18.91 ± 0.04 ^e^	23.68 ± 0.07 ^f^	0.001
n–3	0.57 ± 0.03 ^a^	10.65 ± 0.05 ^b^	3.05 ± 0.02 ^c^	2.34 ± 0.11 ^d^	5.13 ± 0.04 ^e^	6.76 ± 0.09 ^f^	0.001
n–6	2.15 ± 0.04 ^a^	24.23 ± 0.05 ^b^	12.14 ± 0.06 ^c^	6.89 ± 0.07 ^d^	13.78 ± 0.07 ^e^	16.95 ± 0.02 ^f^	0.001
UFA/SFA	0.13 ± 0.02 ^a^	0.86 ± 0.03 ^b^	0.39 ± 0.02 ^c^	0.32 ± 0.01 ^d^	0.44 ± 0.03 ^e^	0.52 ± 0.02 ^f^	0.001
PUFA/SFA	0.03 ± 0.01 ^a^	0.66 ± 0.02 ^b^	0.22 ± 0.02 ^c^	0.12 ± 0.03 ^d^	0.28 ± 0.01 ^e^	0.37 ± 0.02 ^f^	0.001
MUFA/SFA	0.09 ± 0.02 ^a^	0.19 ± 0.02 ^b^	0.18 ± 0.03 ^bc^	0.19 ± 0.02 ^b^	0.17 ± 0.02 ^bc^	0.16 ± 0.02 ^c^	0.772
n–6/n–3	3.85 ± 0.06 ^a^	2.24 ± 0.08 ^b^	4.1 ± 0.05 ^c^	2.90 ± 0.05 ^d^	2.77 ± 0.08 ^d^	2.51 ± 0.05 ^e^	0.001
Polyunsaturated index (PI)	2.34 ± 0.07 ^a^	34.44 ± 0.09 ^b^	13.24 ± 0.05 ^c^	5.77 ± 0.08 ^d^	17.76 ± 0.03 ^e^	22.22 ± 0.09 ^f^	0.001
Atherogenic index (AI)	11.55 ± 0.07 ^a^	1.67 ± 0.04 ^b^	3.45 ± 0.07 ^c^	4.24 ± 0.09 ^d^	3.22 ± 0.09 ^c^	2.80 ± 0.13 ^e^	0.001
Thrombogenic index (TI)	6.78 ± 0.09 ^a^	0.68 ± 0.03 ^b^	1.91 ± 0.02 ^c^	2.16 ± 0.04 ^d^	1.43 ± 0.07 ^e^	1.16 ± 0.06 ^f^	0.001

SE: standard error; ^a–f^: values within a column followed by the different superscript letter are significantly different (*p* < 0.05); SFA: saturated fatty acids; MUFA: monounsaturated fatty acids; PUFA: polyunsaturated fatty acids; UFA: unsaturated fatty acids; n-3: omega-3 fatty acids; n-6: omega-6 fatty acids.

## Data Availability

No new data were created or analyzed in this study. Data sharing is not applicable to this article.
